# Structural insights into the duplex DNA processing of TREX2

**DOI:** 10.1093/nar/gky970

**Published:** 2018-10-24

**Authors:** Hiu-Lo Cheng, Chun-Ting Lin, Kuan-Wei Huang, Shuying Wang, Yeh-Tung Lin, Shu-Ing Toh, Yu-Yuan Hsiao

**Affiliations:** 1 Institute of Bioinformatics and Systems Biology, National Chiao Tung University, Hsinchu, Taiwan 30050, ROC; 2 Department of Biological Science and Technology, National Chiao Tung University, Hsinchu, Taiwan 30068, ROC; 3 Department of Microbiology and Immunology, College of Medicine, National Cheng Kung University, Tainan, Taiwan 70101, ROC; 4 Center of Infectious Disease and Signaling Research, National Cheng Kung University, Tainan, Taiwan 70101, ROC; 5 Institute of Molecular Medicine and Bioengineering, National Chiao Tung University, Hsinchu, Taiwan 30068, ROC

## Abstract

The three prime repair exonuclease 2 (TREX2) is an essential 3′-to-5′ exonuclease that functions in cell proliferation, genome integrity and skin homeostasis maintenance. The abnormal expression level of TREX2 can result in broken chromosome, increased susceptibility to skin carcinogenesis and Psoriasis. However, the molecular mechanisms of how TREX2 binds and processes its natural substrates, dsDNA or chromosomal DNA, to maintain genome stability remain unclear. In this study, we present four new crystal structures: apo-TREX2, TREX2 in complex with two different dsDNA substrates, and TREX2 in complex with a processed dsDNA product. Analysis of the structures reveals that TREX2 stacks with the 5′-terminal of dsDNA by a Leu20-Pro21-Asn22 cluster for precisely trimming the 3′-overhang. In addition, TREX2 specifically interacts with the non-scissile strand of dsDNA by an α-helix-loop region. The unique interaction patterns of the TREX2–dsDNA complex highlight the requirement of long double-stranded region for TREX2 binding and provide evidence of the functional role of TREX2 in processing chromosomal DNA. Moreover, the non-processive property of TREX2 is elucidated by the structure of TREX2–product complex. Our work discloses the first structural basis of the molecular interactions between TREX2 and its substrates and unravels the mechanistic actions of TREX2.

## INTRODUCTION

The maintenance of genome integrity is essential for cell survival and proliferation that requires various stress-induced DNA repair pathways, post-replication repair pathways and DNA proofreading systems. Defects in these systems may cause cell death or cancer. TREX2 (three prime repair exonuclease 2) and its human homologous, TREX1, provide majority of the 3′-to-5′ exonuclease activities for their proofreading function in mammalian cells (1–3). The exonuclease activity of TREX2 is responsible for removing the 3′-mismatched sequence of duplex DNA and is associated with cell proliferation and DNA repair (2,4,5). When a genome is exposed to the toxic stress from UV, TREX2 is upregulated and recruited to the nucleus to access the ends of damaged DNAs by interacting with γH2AX (5,6). Dysfunctional TREX2 leads to reduced cell proliferation (5) and increased susceptibility to drug- or UV-induced skin carcinogenesis (6,7), highlighting the important role of TREX2 in DNA replication and tumor suppression. In addition, TREX2-deficient cells exhibit spontaneous broken chromosomes (8,9), suggesting that TREX2 is crucial for suppressing the formation of broken chromosomes through its involvement in error-free post-replication repair (8,10). However, how TREX2 executes its function on its DNA substrates to maintain the genome integrity still remain elusive.

The unique catalytic property of TREX2 has drawn attention for biomedical and biotechnological applications. TREX2 is a keratinocyte-specific exonuclease and is critical for maintaining skin homeostasis on DNA damage (11). Moreover, TREX2 has been demonstrated as a key player in contributing to the pathogenesis of psoriasis by promoting DNA degradation during keratinocyte death (11). Therefore, TREX2 has been recognized as a promising therapeutic target for the treatment of psoriasis (12). In addition, TREX2 can be applied to increase the frequency of gene disruption targeted by endonuclease because its processing of 3′-ends can prevent the precise rejoining of the DNA breaks by the classical non-homologous end joining pathway to drive mutagenic processing. By coupling TREX2 with a sequence-specific homing endonuclease I-SecI in mutagenic non-homologous end-joining, the gene disruption rate can be enhanced up to 25-fold enhancement (13), suggesting the potential role of TREX2 in genome editing (13,14). Understanding the molecular actions of how TREX2 processes duplex DNA will be the essential to facilitate the development of TREX2-based tools in biomedicine and biotechnology.

TREX2 belongs to the family of DEDDh exonuclease, which was named after the four invariant acidic residues and one general base residue in the active site (15), consisting of more than 17 000 members involved in various DNA and RNA metabolism pathways (15). Most members exhibit preferences for the substrate sequence, length and structure. Among the DEDDh exonucleases, TREX1 (16), TREX2 (2,17) and 3′hExo in mammalian cells (18), Snp in *Drosophila* (19), TTHB178 in *Thermus thermophilus* (20), as well as Exo X (21,22) and RNase T (21) in *Escherichia coli* display a similar substrate preference with the duplex DNA/RNA with 3′-overhang; however, their cellular functions are different. Thus, it is challenging to classify the cellular functions of the DEDDh exonucleases based on the substrate preference and *in vitro* activity assays. Therefore, a new molecular basis for providing the clues to classify and to predict the actual biological role for thousands of DEDDh exonucleases is necessary.

TREX1, a homolog of TREX2 and also a member of DEDDh exonuclease family, comprises an N-terminal nuclease domain and a C-terminal transmembrane domain (23,24). Different from TREX2, TREX1 is distributed in both cytoplasm and nucleus. Cytoplasmic TREX1 is an immune silencing factor that degrades the various cytosolic DNAs to prevent nucleic acid-mediated autoimmunity (25,26). Dysfunctional TREX1 causes inflammation and a numerous of autoimmune diseases (27–29). After exposure to genotoxic stresses, TREX1 only remains its N-terminal nuclease domain and is translocated to the nucleus. The possible functions of nuclear TREX1 include DNA damage-induced replication, DNA proofreading and various DNA repair pathways (1,23). TREX1 and TREX2 share over 40% sequence identity in nuclease domain and both of the them are non-processive exonucleases with similar substrate preference (3,4,17); however, their dsDNA-binding affinity is different (17) and their cellular functions are not overlapped. Thus, structure determination of the TREX2–dsDNA complexes will help us to find the key structural elements that govern the dsDNA-binding preference and to understand the molecular basis of distinct cellular functions of two similar exonucleases.

To date, the three-dimensional structures of TREX2 in complex with duplex DNA are still not available and the molecular mechanisms of how TREX2 binds and processes duplex DNA in various genomic integrity-maintaining pathways remain unclear. In addition, it is not clear how the structural differences in duplex DNA binding mode between TREX2, TREX1 and other DEDDh exonucleases correlate with their functions and natural substrates. Furthermore, the structural basis for the non-processive manner of TREX2 in digesting DNA also remains to be elucidated. In this study, by combining X-ray crystallographic studies on the apo-TREX2, two TREX2–substrate complexes and one TREX2–product complex, and *in vitro* activity assays, we reveal the first molecular mechanisms of TREX2 in binding and processing duplex DNA with 3′-overhang, and provide the structural evidence for the non-processive property of TREX2. Moreover, by structural superposition of TREX2-duplex DNA complex with other DEDDh exonuclease–dsDNA/dsRNA complexes, we provide new structural insights into classifying the DEDDh exonucleases with similar substrate preference, and a framework for predicting the cellular functions of the DEDDh exonucleases.

## MATERIALS AND METHODS

### Protein expression and purification

Mouse TREX2 gene (Gene ID in NCBI: 24102) was cloned into a pET28a vector and expressed in *E. coli* BL21-CodonPlus (DE3)-Rosetta strain. *Escherichia coli* cells were cultured in Luria Broth (LB) medium at 37°C supplemented with 50 mg/ml kanamycin and 35 mg/ml chloramphenicol to an OD_600_ of 0.4–0.6 and then induced with 1 mM isopropyl β-d-1-thiogalactopyranoside at 18°C for 18 h. The cells were harvested through centrifugation and further lysed through sonication in 50 mM Tris–HCl, 300 mM NaCl, pH 8.0. The lysate was clarified through centrifugation at 13 000 rpm at 4°C for 20 min. The supernatant was loaded into an affinity column (HiTrap TALON crude 5 ml, GE Healthcare) and purified by standard protocol. mTREX2 was further purified by an ion-exchange column (HiTrap Q 5 ml, GE Healthcare) and a size-exclusion column (HiLoad 16/60 Superdex 75 prep grade, GE Healthcare). Purified mTREX2 was concentrated to 5–8 mg/ml in 50 mM Tris–HCl, 400 mM NaCl, pH 7.0, and stored at −20°C until use. Active site mutated mTREX2 (H188A) was purified by the same procedure.

### Nuclease activity assays

The sequences of DNA substrates are listed in Supplementary Table S1. DNA substrates were labeled with fluorescein amidite (FAM) at the 5′-end by MDBio, Inc., Taiwan. The labeled substrates (0.5 μM) were incubated with purified mTREX2 at 37°C at 120 mM NaCl, 20 mM Tris–HCl, pH 7.0 and 2 mM MgCl_2_ for 30 min. The reaction was stopped by adding 2 × TBE–urea sample loading buffer (G-Biosciences, USA) at 95°C for 5 min. The DNA digestion patterns were resolved on 20% denaturing polyacrylamide gels and visualized by ultraviolet light. For the polymerase chain reaction (PCR)-generated substrates, we added protease K into the reaction mixture in 37°C for 1 h to stop the reaction. The DNA digestion patterns were resolved on 1% agarose gel and visualized by ultraviolet light.

### Crystallization and crystal structural determination

mTREX2 (5–8 mg/ml) was mixed with different DNA substrates at a molar ratio of 1:1.2. Detailed information regarding DNA sequences and crystallization conditions of the four structures are shown in Supplementary Table S2. All crystals were cryoprotected by Paraton-N (Hampton Research, USA) for data collection at BL13C1, BL13B1 and BL15A1 in NSRRC, Taiwan. All diffraction data were processed by HKL2000, and diffraction statistics are listed in Table 1. Structures were solved by the molecular replacement method, and the crystal structure of *human TREX2* (PDB entry: 1Y97) (4) was used as the search model by MOLREP of CCP4 (30). The models were built by Coot-0.8.1 (31) and refined by PHEXIX-1.9-1692 (32). Diffraction structure factors and structural coordinates were deposited in the RCSB Protein Data Bank with the PDB ID code of 6A45 for the apo-mTREX2, 6A47 for the mTREX2–Y-shaped DNA complex, 6A4B for the mTREX2–duplex DNA complex and 6A46 for mTREX2–product complex.

**Table 1.** Data collection and refinement statistics

Structure	Apo-mTREX2	mTREX2-Y-shaped DNA complex	mTREX2–duplex DNA complex	mTREX2–product complex
DNA in the structure	None	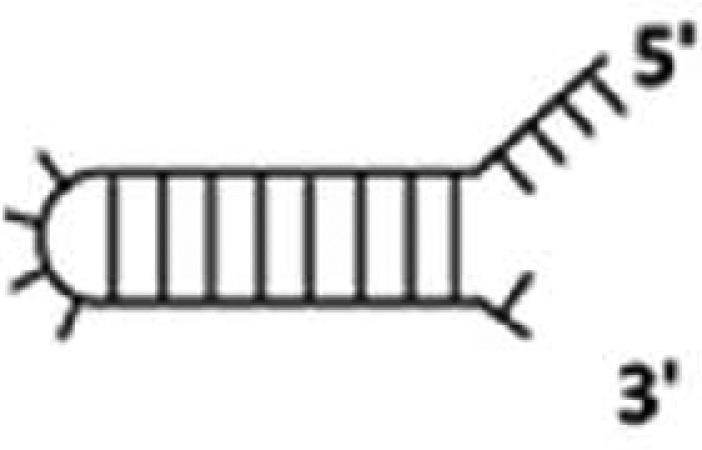	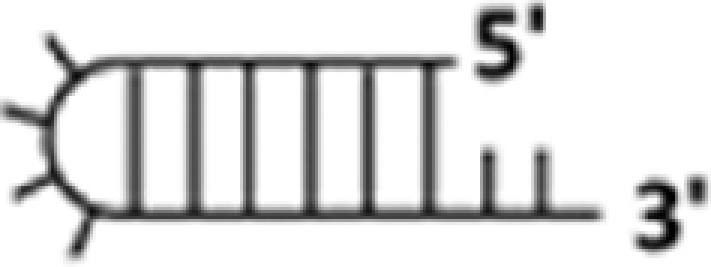	dCMP
Metal ions in one active site	None	2 Mg^2+^	2 Mg^2+^	2 Mg^2+^
**Data collection**				
Space group	P2_1_	C2	P2_1_2_1_2_1_	P2_1_
Cell dimensions				
*a, b, c* (Å)	44.29, 54.73, 88.16	145.66, 48.40, 90.59	48.72, 87.32, 432.94	43.81, 54.77, 88.72
α, β, γ (°)	90.0, 100.7, 90.0	90.6, 90.0, 97.4	90.0, 90.0, 90.0	90.0, 100.8, 90.0
Resolution (Å)	30.0-1.9 (1.97–1.9)	30.0-1.9 (1.97–1.9)	30.0-2.7 (2.8–2.7)	30.0-2.0 (2.07–2.0)
*R*_sym_ or *R*_merge_	6.7 (38.3)	4.3 (45.3)	6.7 (48.6)	5.2 (47.4)
*I* / σ*I*	18.9 (1.8)	33.3 (2.8)	24.2 (2.0)	22.4 (1.9)
Completeness (%)	99.2 (97.3)	99.8 (99.6)	97.1 (97.1)	99.1 (98.1)
Redundancy	3.8 (3.0)	3.9 (3.5)	3.5 (3.1)	3.1 (2.6)
**Refinement**				
Resolution (Å)	26.4-1.9	28.5-1.9	29.2-2.7	28.9-2.0
No. reflections	32319	49670	50754	27789
*R*_work_ / *R*_free_	18.6/21.8	16.1/19.3	21.6/26.0	19.6/22.9
R.m.s. deviations				
Bond lengths (Å)	0.006	0.008	0.003	0.003
Bond angles (°)	0.977	1.118	0.676	0.752
Ramachandran blot statistics (%)				
Favored region	98.1	99.07	96.67	97.65
Allowed region	1.9	0.93	3.25	2.35
Outlier region	0	0	0.08	0

^a^Each structures was obtained from a single crystal.

^b^Values in the parentheses are for highest-resolution shell.

## RESULTS

### Catalytic properties of TREX2: TREX2 prefers to trim the single-stranded 3′-overhang at duplex DNA

**Figure 1.**
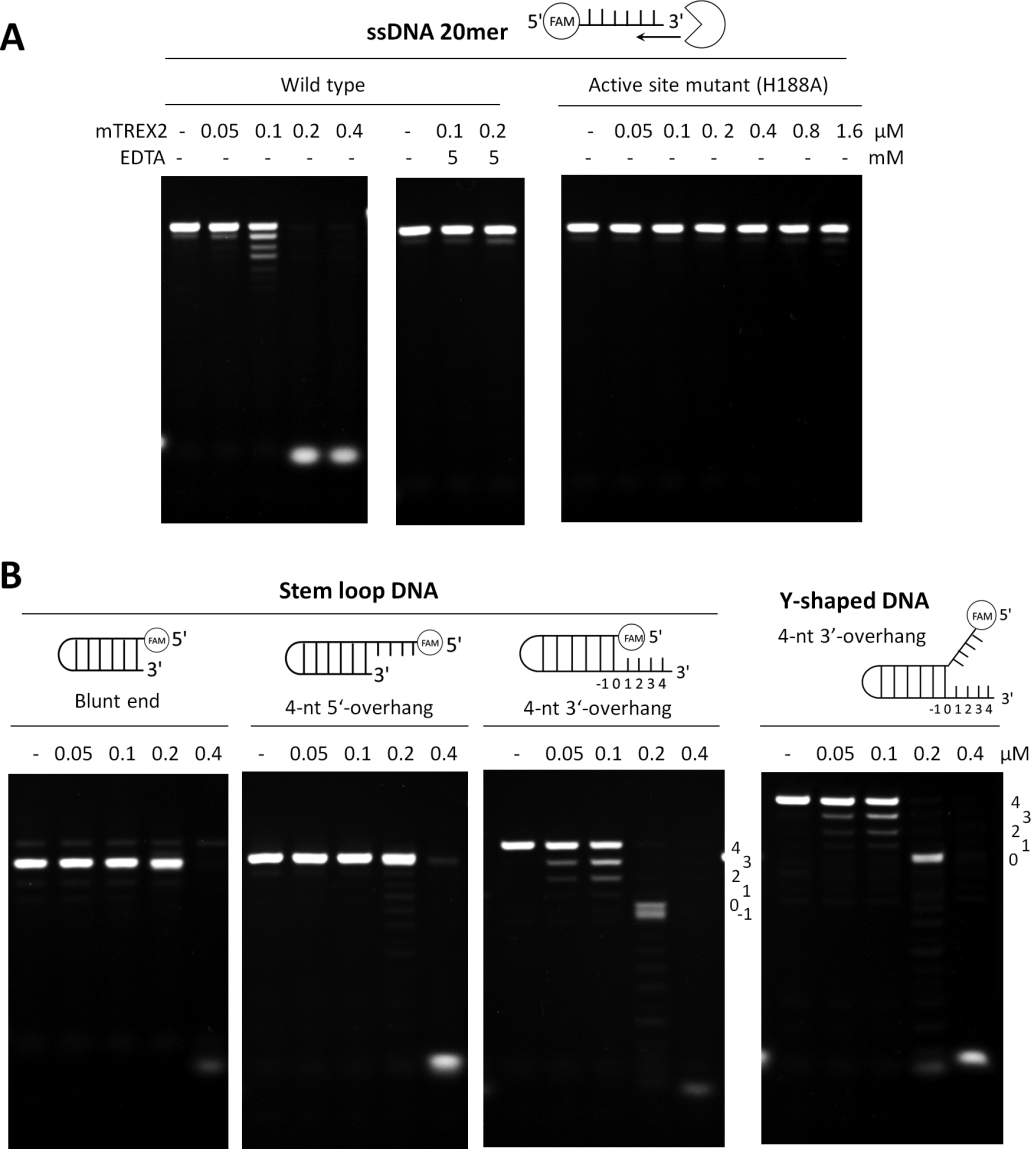
Catalytic properties of mTREX2. (**A**) Nuclease activity assays of mTREX2 on digesting ssDNA (20 mer). ssDNA was fully digested by mTREX2 at the enzyme concentration of 0.2 μM in the presence of 1 mM magnesium ions in the reaction, but did not digested in the presence of 5 mM EDTA. The ssDNA was also not digested by active site mutated mTREX2 (H188A). (**B**) Nuclease activity assays of mTREX2 on digesting duplex DNA (stem loop DNA) with blunt end, 4-nt-long 5′-overhang or 4-nt-long 3′-overhang and Y-shaped DNA. The 3′-overhang at duplex DNA or Y-shaped DNA was removed by mTREX2 at an enzyme concentration of 0.2 μM. The cleavage pattern showed that mTREX2 is blocked by a double-stranded structure. At high enzyme concentration of 0.4 μM, the double-stranded DNA was also fully digested by mTREX2.

To confirm the recombinant mouse TREX2 (mTREX2) protein used in this study is a functional exonuclease that specifically trims the single-stranded 3′-overhang at duplex DNA, we purified mTREX2 (Supplementary Figure S1) for characterizing its catalytic activity. The purified mTREX2 was incubated with the ssDNA substrate and the results showed that the recombinant mTREX2 was able to digest the ssDNA substrate in the presence of magnesium chloride in a concentration-dependent manner. In contrast, the catalytic activity of mTREX2 was inhibited in the presence of 5 mM ethylenediaminetetraacetic acid (EDTA), a metal ion chelating agent, revealing that magnesium ion is essential for the nuclease activity of mTREX2 (Figure 1A). In addition, the ssDNA substrate was not digested by the catalytically inactive mutant mTREX2 H188A, even the concentration of mTREX2 H188A was up to 1.6 μM (Figure 1A). Among the various substrates, including duplex DNA with blunt end, 4-nt-long 5′-overhang, 4-nt-long 3′-overhang and Y-shaped DNA with 4-nt-long 3′-overhang, the lower concentration of mTREX2 (0.2 μM) specifically trims the single-stranded 3′-overhang but not the double-strand structure. Interestingly, mTREX2 was capable of digesting the double-stranded substrate at a higher concentration (0.4 μM) (Figure 1B). Our results showed that TREX2 exhibits the single-stranded DNA-specific activity at a lower concentration and possesses both single- and double-stranded DNA processing activity at a higher concentration that is similar to those of Exo X (21) and TREX1 (16,21), the *E. coli* and human homologs of TREX2 (Supplementary Figure S1). The unique catalytic properties of TREX2, Exo X and TREX1 are different from those of other DEDDh exonucleases, such as *E. coli* RNase T (16,33) which cannot deconstruct the double-stranded DNA structure at the higher concentration of 3 μM (Supplementary Figure S1).

### Crystal structure of apo-mTREX2: TREX2 is a dimeric exonuclease

**Figure 2.**
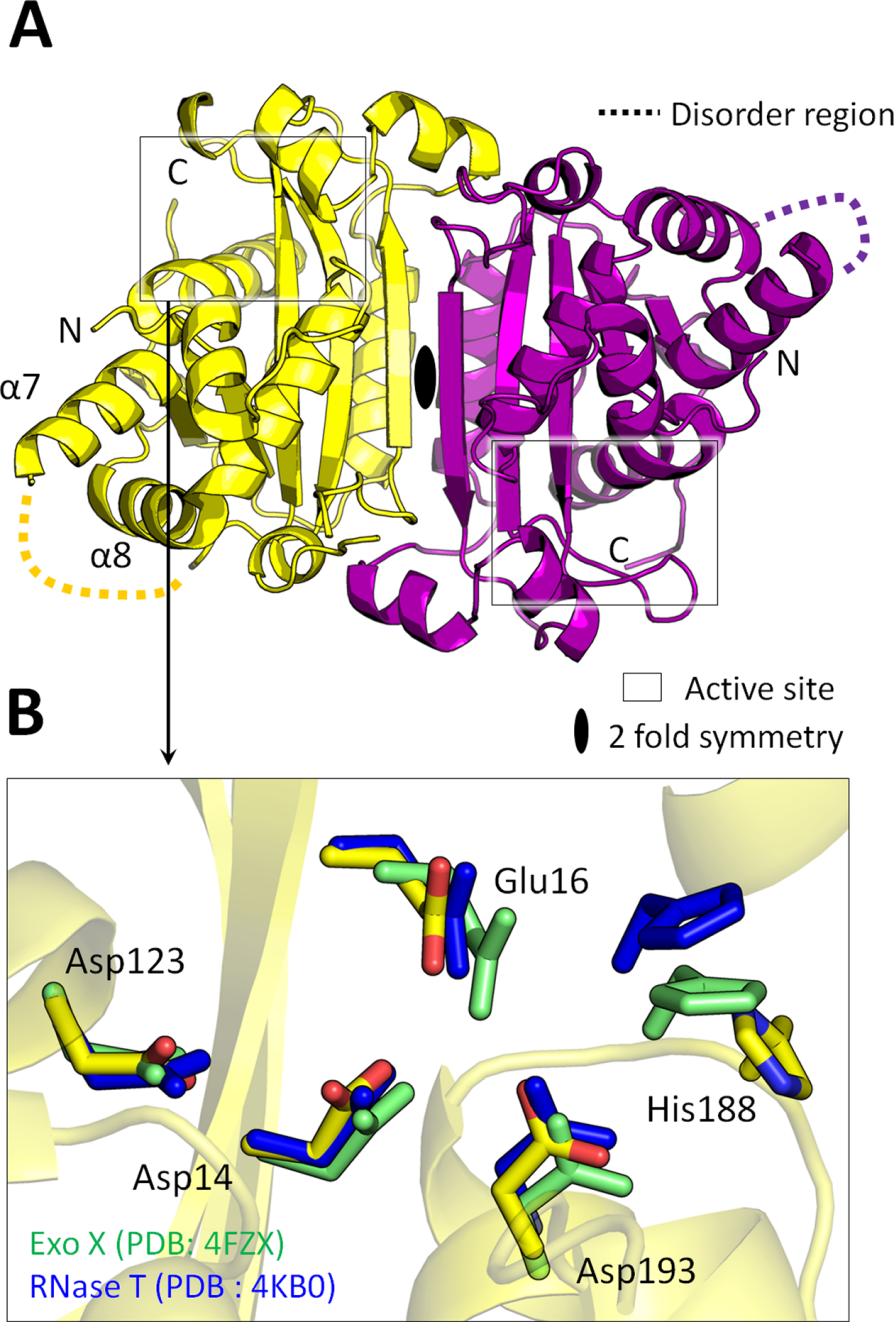
Structure of apo-mTREX2. (**A**) Overall structure of apo-mTREX2. The two TREX2 protomers are colored yellow and purple. The loop region (159 a.a.-167 a.a) between the seventh and eighth α-helix (α7 and α8 helix) is disordered in the structure. (**B**) Active sites superposition of mTREX2, RNase T (PDB entry: 4KB0) and Exo X (PDB entry: 4FZX). The residues of RNase T and Exo X are colored green and blue, respectively.

To provide structural insights into TREX2, we determined four crystal structures: the apo-mTREX2, two TREX2–substrate complexes and one TREX2–product complex. The four structures were solved by molecular replacement using the crystal structure of human apo-TREX2 (PDB entry: 1Y974) or mouse apo-TREX2 (PDB entry: 6A45) as the searching model. The details of crystallization conditions, diffraction and refinement statistics are listed in Table 1 and Supplementary Table S2. The crystal structure of apo-mTREX2 was solved at 1.9 Å resolution. The structure reveals the assembly of a TREX2 dimer in the asymmetric unit, with a two-fold symmetry axis at the center of the dimer interface, and the homodimeric conformation is consistent with the solution state of mTREX2 by gel-filtration analysis (Figure 2 and Supplementary Figure S1). Formation of the dimer is important for the cooperative DNA binding and the exonuclease activity of TREX proteins, such as TREX1 and TREX2 (3,34–36). Each monomer only consists of protein molecule without any metal ions. The residues of the active site, Asp14, Glu16, Asp123, Asp193 and His188, aligned well with those of *E. coli* RNase T and Exo X which are the typical members of DEDDh exonucleases, suggesting their structural conservation (Figure 2). The absence of metal ions in the structure of apo-mTREX2 suggests the metal ions are stabilized upon the binding of substrate DNA to mTREX2 during catalytic process.

### Crystal structure of mTREX2-Y-shaped DNA complex: the scissile and non-scissile strands of duplex DNA are required for TREX2 binding

**Figure 3.**
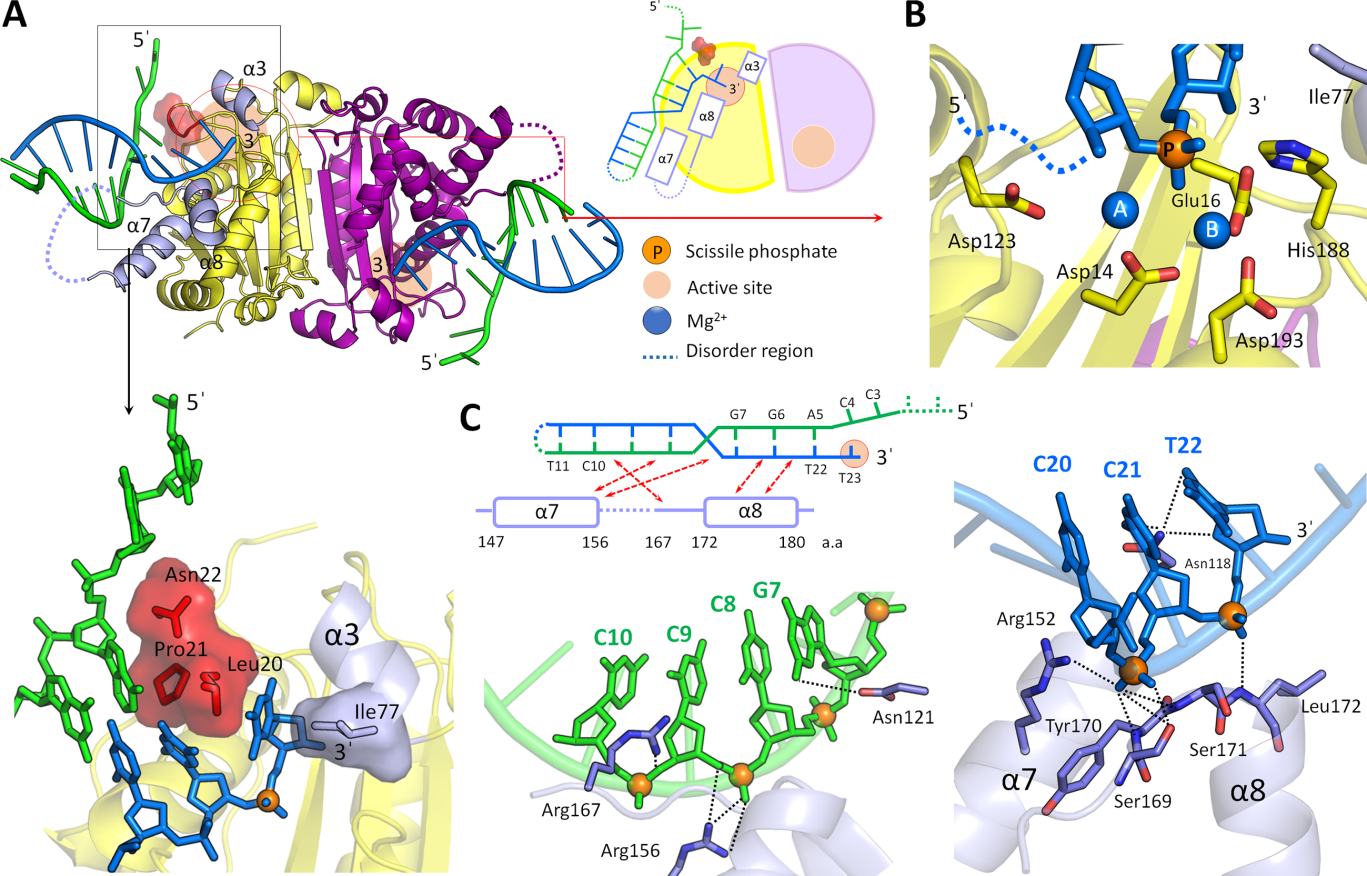
Structure of mTREX2-Y-shaped DNA complex. (**A**) Overall structure, schematic diagram, and DNA termini binding region of mTREX2-Y-shaped DNA complex. The scissile and non-scissile strands of Y-shaped DNA are colored blue and green, respectively. The DNA termini stacking cluster is constructed by Leu20, Pro21 and Asn22, which are colored red and displayed by stick and surface modes. Three DNA binding related α-helices (α3, α7 and α8) are colored in light purple. (**B**) The active site structure of mTREX2-Y-shaped DNA complex. The orange and blue balls are scissile phosphate and two magnesium ions. (**C**) DNA interacting regions of scissile and non-scissile strands of Y-shaped DNA. The orange balls are phosphates in the Y-shaped DNA.

To provide structural insights into the duplex DNA processing by TREX2, we determined crystal structures of mTREX2-Y-shaped DNA complex. The omitted electron density map of the Y-shaped DNA fits well to the three-dimensional structure (Supplementary Figure S2). In the complex structure, one mTREX2 dimer is associated with two Y-shaped DNAs in an asymmetric unit of the mTREX2-Y-shaped DNA complex structure. The Y-shaped DNA is a 23-nt-long stem loop DNA with unpaired 1-nt-long 3′-overhang and 4-nt-long 5′-overhang. The 1-nt-long 3′-overhang of the two Y-shaped DNAs are deeply inserted into the active sites of mTREX2 dimer and stacked by residues Leu20 and Ile77. The last two bases of 4-nt-long 5′-overhang are disordered and cannot be visualized in the structure. In addition, the C12 and T13 bases in the loop region of Y-shaped DNA and the residues located at loop region between the seventh and eighth α-helix (residues 160–166) of mTREX2 protein are also disordered in the complex structure (Figure 3A).

The 3′-ended four bases of the scissile strand (C20 to T23) of Y-shaped DNA dominantly interact with mTREX2 to form numerous hydrogen bonds with the side chain and/or main chain of Asp14, Glu16, Ala17, Leu20, His117, Asn118 and residues located at the α7, α8 and the loop between α7 and α8 helices (α7/α8 loop) (Supplementary Figure S3). The tight hydrogen-bonding network between the last four bases of scissile strand and mTREX2 are commonly observed in structures of other DEDDh exonuclease–substrate complexes. In contrast, the binding of mTREX2 by residues Asn22, Asp121, Arg156 and Arg167 to the sugar–phosphate backbone of the non-scissile strand is unique among DEDDh exonucleases (Figure 3C). A previous mutational study indicates that the R156L mutant showed a 6.5-fold lower activity for dsDNA degradation than the wild-type TREX2, confirming the role for binding to the non-scissile strand DNA of Arg156 (6). In addition, mutation at R167 also affect the DNA-binding and nuclease activity of TREX2 (5,9,34). These observations indicate that the DNA-binding residues identified in our structures are consistent with previous biochemical studies (4,5,34). The similar hydrogen-bonding network between mTREX2 and sugar–phosphate backbone of the non-scissile strand is also observed in a few members of DEDDh exonucleases, such as RNase T and Exo X whose nature substrates are dsRNA (37,38) and dsDNA, respectively (22,39). This finding suggests that the nature substrate of TREX2 is also a double-stranded DNA. In order to confirm the ability of TREX2 in dsDNA degradation, and the roles of Arg156 and Arg167 in dsDNA binding, we generated substrates by PCR for nuclease activity assay, which was the previously used method for Exo X and Exo X mutants (22). Our results clearly shown the PCR product was fully digested by wild-type mTREX2 at the enzyme concentration of 1.6 μM. In contrast, the PCR product was not fully digested by the catalytically inactive mutant mTREX2 H188A. In addition, the binding site mutants, mTREX2 R156A, R167A and R156A/R167A, shown reduced nuclease activity at the same enzymatic concentration (Supplementary Figure S4). These experiments confirmed that two residues, Arg156 and Arg167, identified from the structure of the TREX2-Y-shaped DNA complex are important for the activity of dsDNA digestion of TREX2.

### Molecular mechanism for TREX2 precisely trimming the 3′-overhang

The structural origin of TREX2 precisely trimming the single-stranded 3′-overhang of duplex DNA and the possible final product of Y-shaped DNA digested by TREX2 can be revealed by the mTREX2-Y-shaped DNA complex structure. First, the active sites of mTREX2-Y-shaped DNA complex adopt an active form conformation with the presence of two magnesium ions (Figure 3B). When TREX2 binds to the Y-shaped DNA with 1 nt-long 3′-overhang, TREX2 can remove the last nucleotide at the 3′-overhang to generate a duplex DNA without 3′-overhang. Second, the last base pair (A5-T22 base pair) of Y-shaped DNA is stacked by a cluster constructed by Lue20, Pro21 and Asn22 (Figure 3A); among which, the sugar ring of A5 also interacts with Asn22 by a hydrogen bond. These interactions construct a steric hindrance to block the exonuclease activity of TREX2 and consequently prevent dsDNA from being further digested by TREX2. Hence, the final product of duplex DNA with 3′-overhang digested by TREX2 is duplex DNA without 3′-overhang. In summary, the structure of the active site in the active form of TREX2 and the steric hindrance for the double-strand structure of dsDNA confirm the precise removal of the single-stranded 3′-overhang and the suspension at the double strand region of duplex DNA by TREX2. These findings are also consistent with our biochemical studies (Figure 1).

### Crystal structure of mTREX2–duplex DNA complex: display the similar dsDNA-binding mode to mTREX2-Y-shaped DNA complex

**Figure 4.**
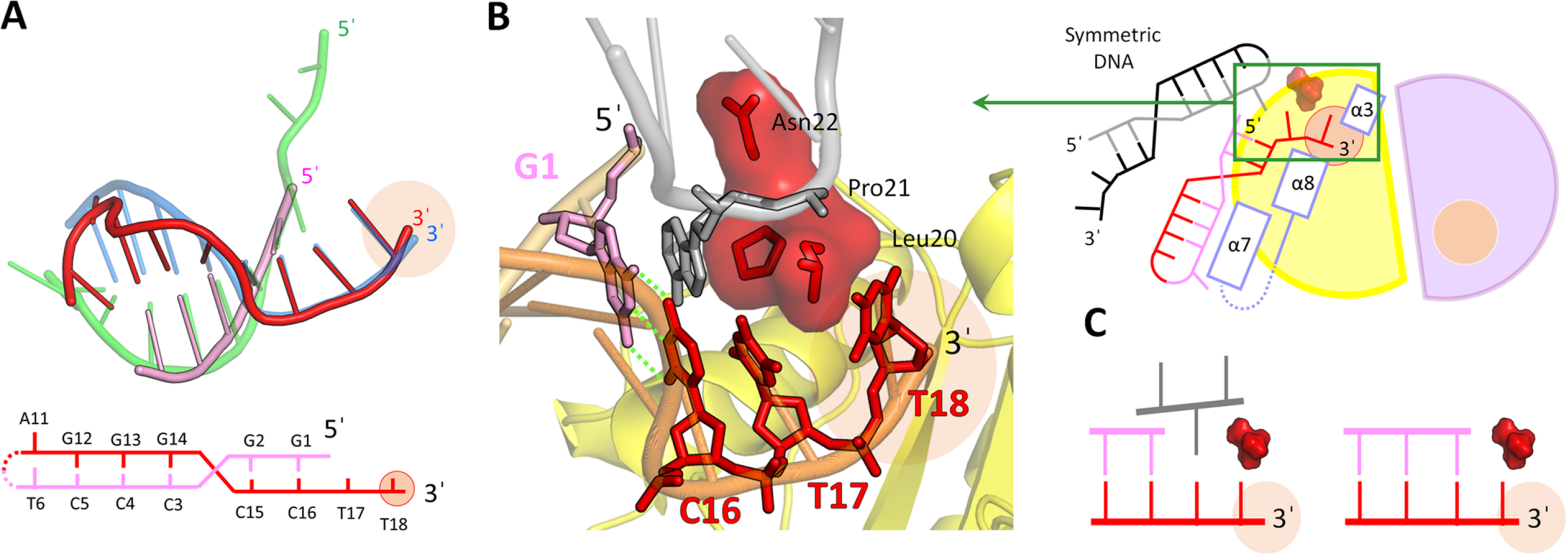
Structure and structural comparison of mTREX2–duplex DNA complex. (**A**) DNA structural comparison of mTREX2–duplex DNA complex and mTREX2-Y-shaped DNA complex. The scissile strand and non-scissile strand of duplex DNA in mTREX2–duplex DNA complex are colored red and pink, respectively. The bottom panel shows the DNA sequence of duplex DNA. The Y-shaped DNA in mTREX2-Y-shaped DNA complex is displayed in transparent mode and colored blue and green. (**B**) Close view of the DNA terminal binding region of mTREX2. The DNA termini stacking cluster are colored red and displayed by stick and surface modes. The DNA from other asymmetric unit is colored gray or black. (**C**) Schematic comparison of the DNA terminal of mTREX2–duplex DNA complex and mTREX2-Y-shaped DNA complex structures. The active sites are displayed as pink balls.

To confirm the molecular mechanism for TREX2 precise trimming the 3′-overhang of duplex DNA, we determined a structure of TREX2–duplex DNA complex at a resolution of 2.7 Å. The sequence and structure of duplex DNA with 2-nt-long 3′-overhang are shown in Figure 4A. The DNA-binding mode of a duplex DNA with 3′-overhang bound by TREX2 is similar to that of Y-shaped DNA. Based on the superposition of the two structures of TREX2–substrate complexes, we observed that the regions of double-stranded and 3′-overhang in the two DNAs are thoroughly overlapped (Figure 4A). The active sites of TREX2 in the complex also adopt an active form structure. These two structures indicate the similarity of DNA-binding mode of duplex DNA to the short 3′-overhang bound by TREX2.

In the structure of mTREX2–duplex DNA complex, the gap between the last base at the double-stranded region of duplex DNA (G1 base) and the Leu20-Pro21-Asn22 cluster of TREX2 is filled up by a base at the loop region of symmetric duplex DNA (Figure 4B and C). This binding mode is similar to that of Y-shaped DNA bound by TREX2 in the TREX2-Y-shaped DNA structure as shown in Figure 4C. Therefore, for duplex DNA and Y-shaped DNA substrates containing a short 3′-overhang, the pattern of specific interactions is similar between TREX2 and the terminal of duplex DNA, including 5′-end capping by the Leu20-Pro21-Asn22 cluster. In both cases, a duplex DNA without 3′-overhang is the expected final product consistent with the nuclease activity assay (Figure 1).

### Crystal structure of mTREX2–product complex: reveal the molecular mechanism of non-processive DNA processing mode of TREX2

**Figure 5.**
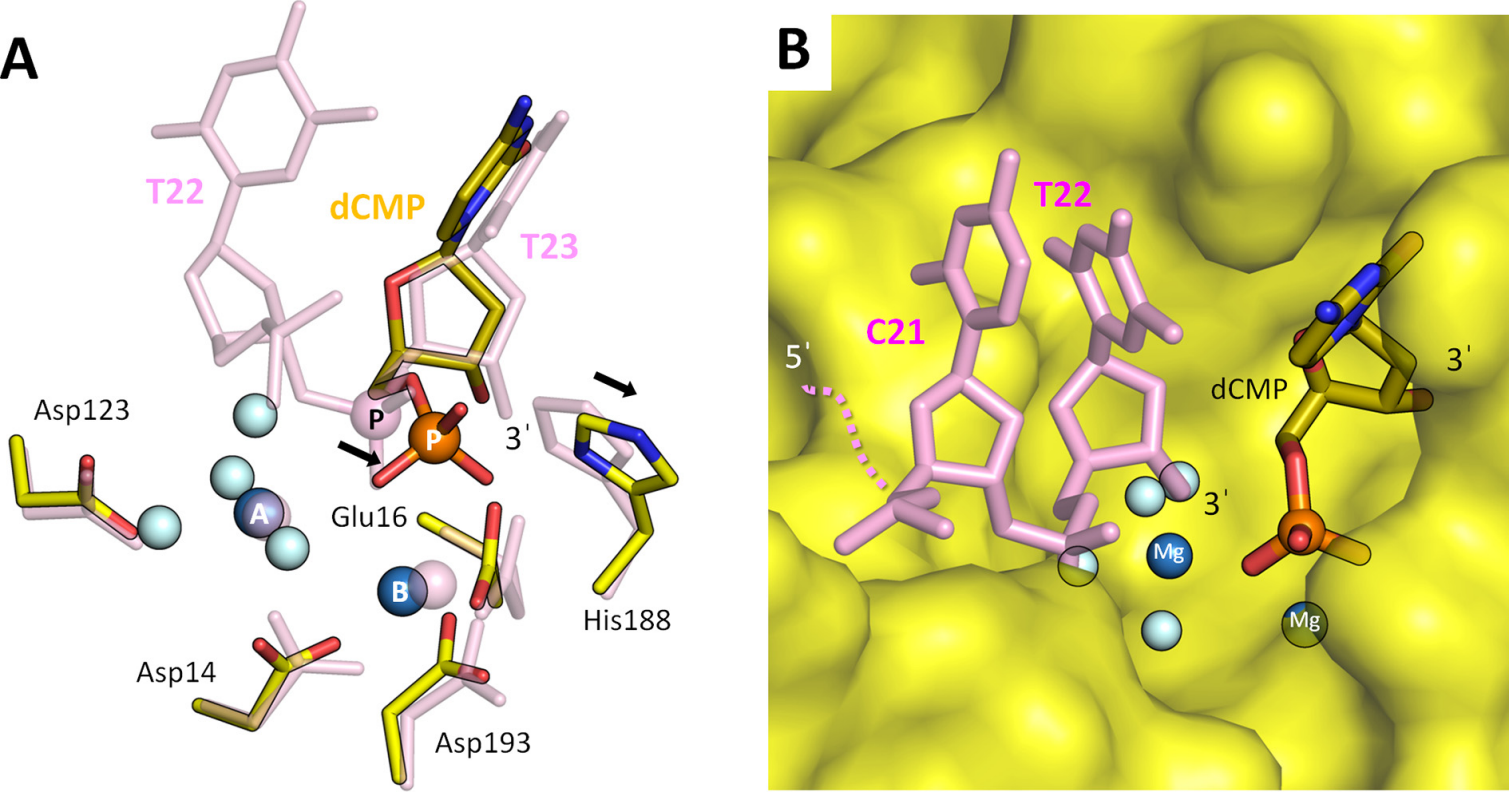
Active site structure of mTREX2–product complex. (**A**) The superposition of the two active sites in mTREX2–substrate complex (mTREX2-Y-shaped DNA complex) and mTREX2–product complex. The substrate complex is colored pink. The scissile phosphates are displayed as pink or orange balls and labeled ‘P’. The water molecules and two Mg^2+^ are displayed as light blue and blue balls, respectively. (**B**) Structural model of the transition state is between substrate cleaving and product releasing. mTREX2 protein is displayed as surface mode.

TREX2 is a well-known non-processive exonuclease and identified by isotope-based nuclease activity assay in previous studies (4,17). In order to understand the non-processive mechanism of TREX2 in DNA processing, we determined mTREX2–product complex. In the complex, the input substrate DNA is degraded into single nucleotides by mTREX2 during crystallization. The details of crystallization conditions listed in Supplementary Table S2. The final structure includes the mTREX2 dimer with two magnesium ions (MgA and MgB) and one product, deoxycytidine monophosphate (dCMP), per active site. MgA and MgB adopt a classic hexa-coordination pattern and a non-canonical penta-coordination pattern, respectively. The omitted electron density map (*σ* = 2.0) fits perfectly with the three-dimensional structure of magnesium ions and dCMP (Supplementary Figure S5A). The orientation of magnesium ions, dCMP and active site residues are consistent with the structures of other DEDDh exonuclease in complex with products, such as TREX1–product complex (Supplementary Figure S5B). The structural studies on TREX2–product complex provides the molecular origin of the non-processive mode of TREX2 in digesting DNA substrates. According to the structural comparison of the substrate complex, mTREX2-Y-shaped DNA complex, the scissile phosphate and general base are shifted; specifically, the scissile phosphate is moved into the narrow groove in the active site (Figure 5A and B). Furthermore, we generated a structural model to mimic the transition state between substrate cleaving and product releasing (Figure 5B). The model is composed of TREX2–product complex and a cleaved dsDNA substrate. The cleaved dsDNA substrate is generated from the removed 3′-ended nucleotide (T23 base) Y-shaped DNA in mTREX2-Y-shaped DNA complex which blocks the entrance of the groove (Figure 5B). Hence, the product (dCMP) cannot be released from the narrow groove in the active site and the cleaved DNA substrate cannot be further processed by TREX2. Therefore, the cleaved DNA substrate should be released before the product; in the other words, the DNA substrate cannot be continuously processed by TREX2.

## DISCUSSION

### The molecular mechanism of TREX2 in duplex DNA processing

Our structural and biochemical analyses demonstrate that TREX2 contains a catalytic DEDDh exonuclease domain, which binds and processes duplex DNA in a unique way. TREX2 removes the single stranded 3′-overhang precisely and the processing is blocked by double strand structure resulted in dsDNA product without 3′-overhang. Our determined crystal structures of mTREX2-Y-shaped DNA complex and mTREX2–duplex DNA complexes, clearly showed the Leu20-Pro21-Asn22 cluster is responsible for stacking with the 5′-end of duplex DNA to control the precise trimming process. The abounded hydrogen bonds in these two structures formed between TREX2 and non-scissile strand of duplex DNA required, suggesting the nature substrates of TREX2 are longer double-stranded DNA. In addition, the structure of mTREX2–product complex demonstrates the narrow groove in the active site limits the product releasing (nucleoside monophosphate); thus, the cleaved DNA substrate should be released before the product, which provides the structural evidence for the non-processive property of TREX2.

### Structural difference between TREX1 and TREX2 in binding to duplex DNA

**Figure 6.**
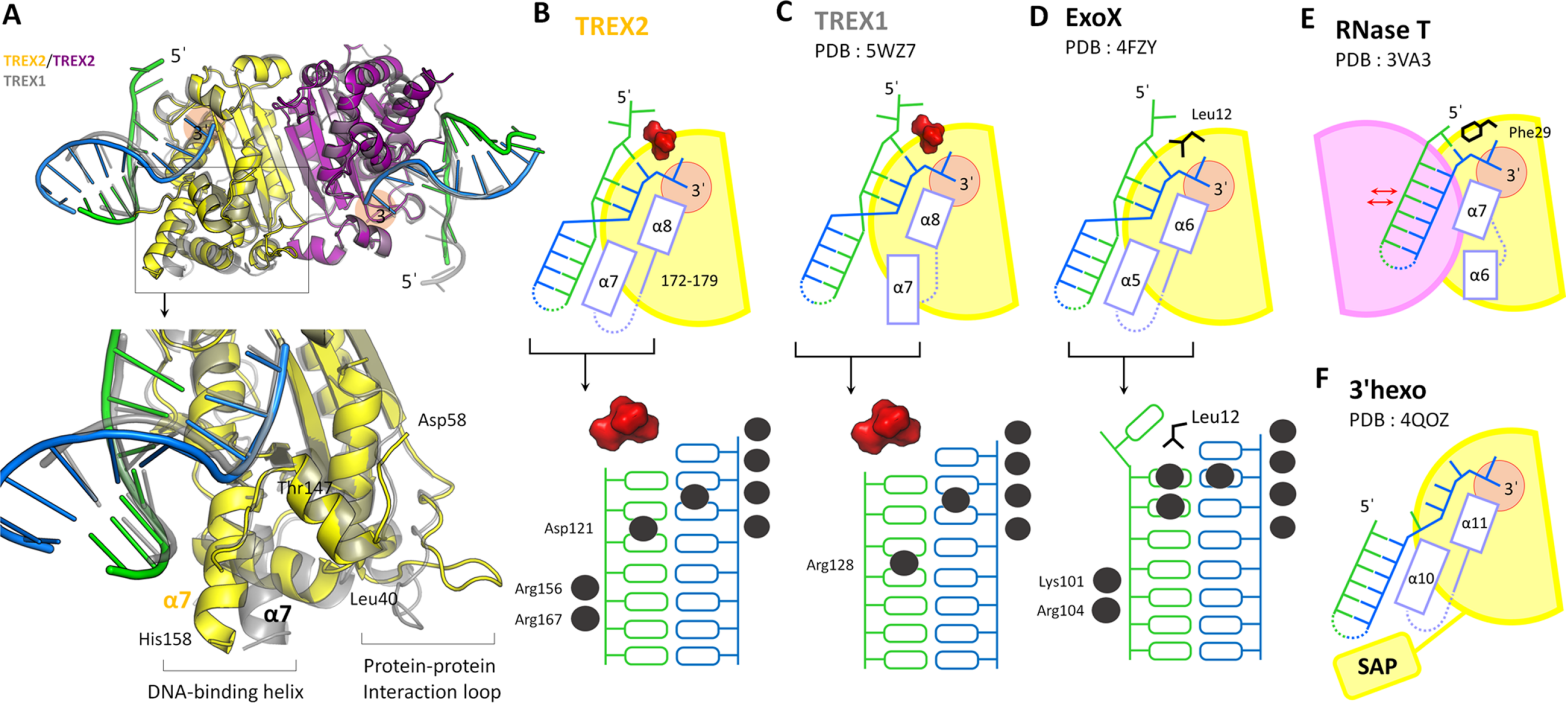
Structural difference between TREX2 and other DEDDh exonucleases in DNA binding. (**A**) Structural comparison of mTREX2-Y-shaped DNA complex and TREX1-dI-T–dsDNA complex. The DNA and protein of TREX1-dI-T–dsDNA complex are colored gray. (**B**–**F**) Schematic diagram of TREX2 and other DEDDh exonucleases in complex with duplex DNA/RNA. The active sites in these structures are displayed as orange circles. The lower panel shows the protein–DNA interacting pattern of various DEDDh exonuclease–DNA complexes. The interaction between protein and DNA are marked by black balls. SAP in (F) is the SAP domain in 3′hExo.

TREX1 and TREX2 provide majority of the 3′-to-5′ exonuclease activity in mammalian cells (1–3). The amino acid sequence identity of the nuclease domain of TREX1 and TREX2 is higher than 40%; therefore the secondary structure, the overall three-dimensional structure and the dimer orientation are also similar as expected (Figure 6A and Supplementary Figure S6A). However, these two TREX proteins have different DNA-processing-related cellular functions and DNA binding affinity (17). One possible explanation is that TREX1 contains an extended 19 amino acid-long proline-rich loop (residues 45–64), which is responsible for mediating protein–protein interaction (Figure 6A and Supplementary Figure S6) (3,40). The different interacting partners may define the distinct cellular functions and DNA-binding affinity of these two TREX proteins. However, the proline-rich loop of TREX1 does not interact with DNA and does not seem to directly affect the DNA binding; the structural features for the different DNA-binding modes of the two similar TREX proteins remain to be elucidated. Based on the structural superposition of two TREX protein–duplex DNA complexes, we observed the key structural differences between TREX1 and TREX2 for DNA binding (Figure 6A). The duplex DNAs bound by TREX2 and TREX1 are superimposable with each other (Figure 6A and Supplementary Figure S6B). The α7 helix (residues 147–158), α8 helix (residues 172–180) and the α7/α8 loop (residues 159–171) of TREX2 are responsible for duplex DNA binding. Among these regions, α7 helix and the α7/α8 loop play major roles in binding to the non-scissile strand, especially at the remote region (Figures 3C and 6B). We also analyzed three structures of TREX1 in complex with duplex DNA with short 3′-overhang, including TREX1-dI-T–dsDNA complex (PDB entry: 5YWU), TREX1-L-shaped–dsDNA complex (PDB entry: 5YWT), and TREX1-Y-shaped–dsDNA complex (PDB entry: 5YWS). The α7 helix (residues 155–165) in the three structures of TREX1–substrate complexes is not involved in the DNA association (Figure 6A and C). Structural analysis revealed that an additional α-helix and loop region contribute to the interaction with the non-scissile strand duplex DNA in TREX2–substrate complex compared with TREX1–substrate complex.

The protein–DNA interaction patterns also highlight the difference of TREX1 and TREX2 in dsDNA binding. In TREX1, only Arg128 commonly interacts with the non-scissile strand of duplex DNA (Figure 6C). In TREX2, Asp121, Arg156 and Arg167 contribute to the binding for the non-scissile strand of duplex DNA ranged over six base pairs in length (Figure 6B). The interacting area for TREX2 on the non-scissile strand of duplex DNA is longer than that for TREX1. The structural features reflect the different cellular functions of TREX1 and TREX2. The structure of TREX2 for binding to duplex DNA with long double-stranded region; thus, TREX2 is suitable for participating in the process of duplex DNA with a longer double-stranded region, such as chromosomal rearrangement and maintenance. The interacting region between TREX1 and duplex DNA focuses on the terminal of duplex DNA; thus, TREX1 fits for dsDNA terminal deconstruction in immune controlling and trimming of structural DNAs in various DNA repair pathways (16). In summary, our results highlight the characteristic of TREX2 on the processing of duplex DNA with long double-stranded region.

### Structural classification of DEDDh exonucleases with specific substrate preference on duplex DNA with 3′-overhang

**Figure 7.**
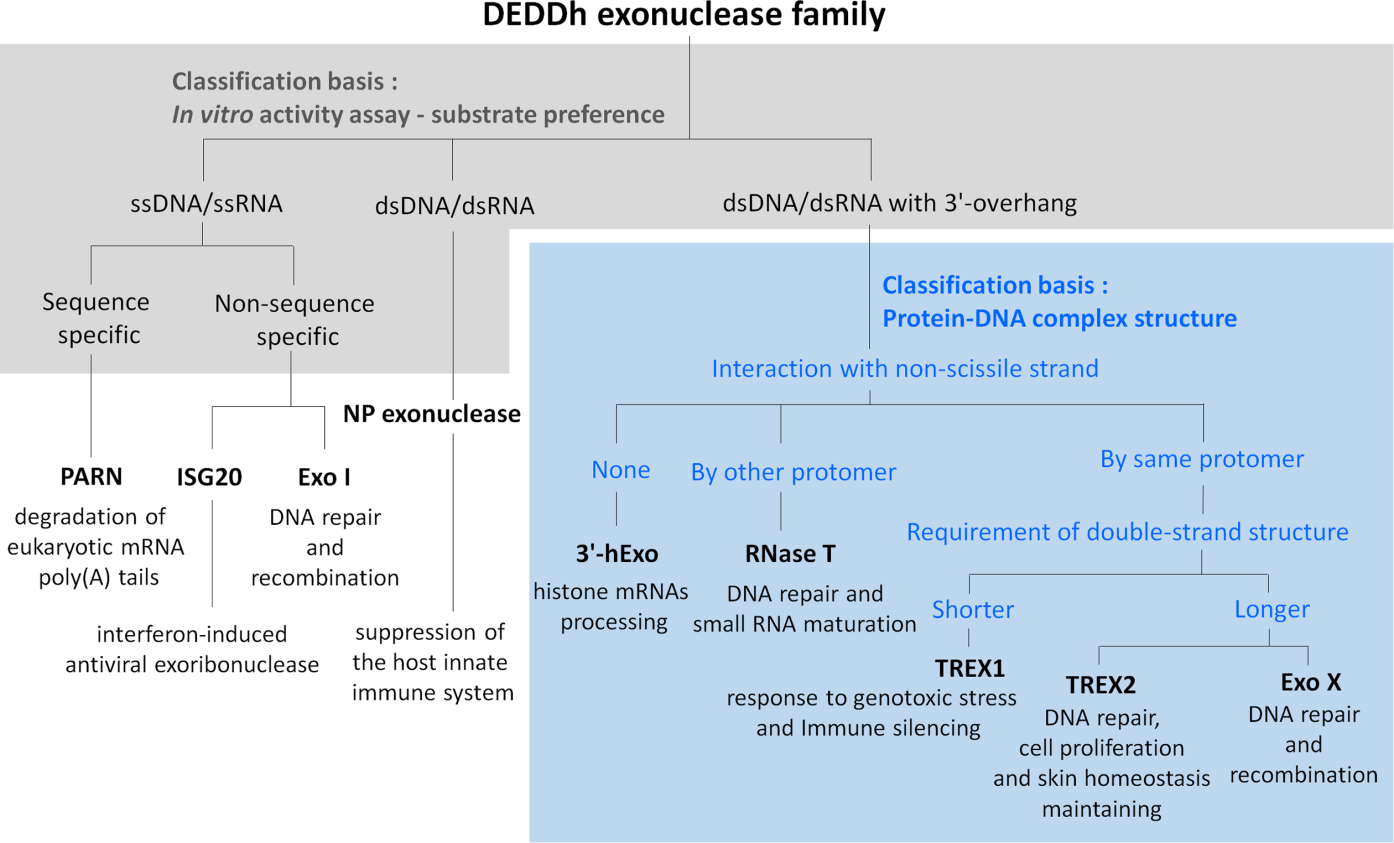
Classification of classical DEDDh exonucleases. DEDDh exonucleases can be classified based on the *in vitro* activity assays (substrate preference) and the structural characteristic in binding to duplex DNA (the interaction to non-scissile strand of duplex DNA and the length requirement of double-stranded region). PARN, poly(A)-specific ribonuclease (43,45); ISG20, Interferon-stimulated gene 20-kDa protein (46); Exo I, *Escherichia coli* Exonuclease I (44,47); NP exonuclease, Lassa Virus NP Exonuclease (49,50); 3′hExo, human 3′-5′ exonuclease, also known as Eri-1 (18,51); Exo X, *E. coli* exonuclease X (22,39,52,53).

The family of DEDDh exonucleases contains over 17 000 members across more than 3000 species from prokaryotes to eukaryotes. More than one DEDDh exonucleases are usually found in one species. For example, over four members are found in *E. coli* and more than 30 members in *Homo sapiens* (in Pfam database: https://pfam.xfam.org/). The studies for the actual cellular functions of these DEDDh exonucleases are stagnant due to their similar substrate preference and the overlapping functions to other exonucleases. In mammalian cells, at least three DEDDh exonucleases, such as TREX1, TREX2 and 3*′*hExo, prefer duplex DNA with 3′-overhang as substrates. The *E. coli* RNase T functionally overlaps other exonucleases in RNA maturation, and single-gene knockout cannot provide an observable phenotype (21,38,41). Therefore, the true cellular functions of specific DEDDh exonuclease are difficult to study only by *in vitro* activity assays and *in vivo* genetic knockout experiments. *Drosophila* Snp and *Caenorhabditis elegans* CRN-4 are the two unsuccessful examples used to define their real cellular functions by *in vivo* and *in vitro* studies in normal cells (19,42). Our structural observation provides a new direction for classification of DEDDh exonucleases, especially for members preferring duplex DNA with 3′-overhang as a substrate (Figures 6 and 7).

Based on the substrate preference measured by *in vitro* activity assays, we divided the DEDDh exonucleases into three groups, including ssDNA/ssRNA-specific (43–47), dsDNA/dsRNA-specific (48–50) and duplex DNA/RNA with 3′-overhang-specific DEDDh exonucleases (16,18,22,39,51–53) (Figures 6 and 7). The 3′-overhang-specific DEDDh exonucleases usually contain a narrow active site, and use two hydrophobic resides to stack with the last base at 3′-end (33,54). The structural features allow these nucleases specifically trim the single-stranded 3′-overhang of duplex DNA. According to the interaction between protein and the non-scissile strand of duplex DNA/RNA, the group of duplex DNA/RNA with 3′-overhang-specific DEDDh exonuclease can be further classified into three subgroups. The representative member of the first subgroup is 3′hExo, which only interacts with the scissile strand of duplex RNA. The representative member of the second subgroup is RNase T, which interacts with the non-scissile strand of duplex DNA/RNA by another protomer of dimeric RNase T. TREX1, TREX2 and Exo X belong to the third subgroup, which interact with both scissile and non-scissile strands of duplex DNA by the same protomer. These three exonucleases specifically trim the 3′-overhang at a low enzymatic concentration and have ability to degrade dsDNA at a high enzymatic concentration (16). The Leu24-Pro25-Ser26 cluster of TREX1, the Leu20-Pro21-Asn22 cluster of TREX2 and a wedge residue Leu12 of Exo X are used to stack or to unwind the double stranded structure for dsDNA processing (16,22). TREX1, TREX2 and Exo X can be further categorized based on the requirement for the double-stranded region on duplex DNA. TREX2 and Exo X require longer double-stranded regions for binding to duplex DNA (22) (Figure 6B and D), suggesting that their natural substrates and cellular functions are more similar to each other than that of TREX1. Thus, TREX2 and Exo X may act on chromosomal DNA and execute their functions at UV-related DNA repair, DNA recombination and genome integrity maintaining (22, 39, 52, 53). The structural framework provided in this study is useful for classifying the DEDDh exonucleases with similar substrate preference and for predicting the cellular roles of new DEDDh exonucleases with unknown functions.

## DATA AVAILABILITY

Coordinates and structure factors were deposited in Protein Data Bank under accession code 6A45 (apo-mTREX2), 6A47 (mTREX2–Y-shaped DNA complex), 6A4B (mTREX2–duplex DNA complex), and 6A46 (mTREX2–product complex).

## Supplementary Material

Supplementary Data
