# Supplementary material for: Structural insights into the duplex DNA processing of TREX2
Source: Nucleic Acids Res. 2018 Oct 24;46(22):12166–76. doi: 10.1093/nar/gky970 (PMC6294518; doi:10.1093/nar/gky970)
Supplement: Supplementary Data [file gky970_supplemental_files.pdf]

Supplementary information

for

Structural insights into the duplex DNA processing of TREX2

Hiu-Lo Cheng[1], Chun-Ting Lin[2], Kuan-Wei Huang[2], Shuying Wang[3,4], Yeh-Tung Lin[2], Shu-Ing Toh[2,5] and Yu-Yuan Hsiao[1,2,5*]

[1]Institute of Bioinformatics and Systems Biology, National Chiao Tung University, Hsinchu, 30050, Taiwan.
[2]Department of Biological Science and Technology, National Chiao Tung University, Hsinchu, Taiwan 30068, ROC.
[3]Department of Microbiology and Immunology, College of Medicine, National Cheng Kung University, Tainan, Taiwan
[4]Center of Infectious Disease and Signaling Research, National Cheng Kung University, Tainan, Taiwan.
[5]Institute of Molecular Medicine and Bioengineering, National Chiao Tung University, Hsinchu 30068, Taiwan

Supplementary Tables: 1-2
Supplementary Figures: 1-6

Supplementary Table 1. Substrates for biochemical studies

**Substrate**	**Sequence**
ssDNA 20 nt	5′- ACTGGACAAATACTCCGAGG -3′
Stem-loop DNA (blunt end)	5′- GGCCCTCTTTAGGGCC -3′
Stem-loop DNA with 5′-overhang (nt)	5′- TTAAGGCCCTCTTTAGGGCC -3′
Stem-loop DNA with 3′-overhang (4nt)	5′- GGCCCTCTTTAGGGCCTTGG -3′
Y structural DNA (3′- and 5′-overhang : 4nt)	5′- TTAAGGCCCTCTTTAGGGCCAAGG -3′

*****The underline regions are the paired region or stem region of DNA substrates.

Supplementary Table 2. Crystallization conditions of apo-mTREX2, mTREX2-substrate DNA complexes and TREX1-product complex

Apo-mTREX2		
Input DNA : None<br>Time of growth: 4 weeks<br>Condition: 0.1 M HEPES sodium pH 7.5, 0.8 M Sodium phosphate monobasic monohydrate, 0.8 M Potassium phosphate monobasic<br>Activity of TREX2 in crystallization condition: Inhibited	Input DNA	DNA in the structure
	None	None
**mTREX2-Y-shaped DNA complex**		
Input DNA[a]: 5′-TGCC**AGGCCCTCTTTAGGGCCT**T-3′<br>Time of growth: 2-3 weeks<br>0.1 M Sodium citrate tribasic dihydrate pH 5.0, 30% v/v Polyethylene glycol monomethyl ether 550, D-(+)-Glucose monohydrate<br>Activity of TREX2 in crystallization condition: Inhibited	Input DNA	DNA in the structure[b]
	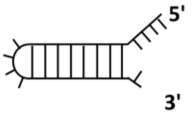	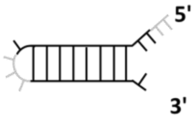
**mTREX2-duplex DNA complex**		
Input DNA[a]: 5′-**GGCCCTCTTTAGGGCC**TT-3′<br>Time of growth: 3-5 weeks<br>Condition: 0.1 M Sodium citrate tribasic dihydrate pH 5.5, 22% w/v Polyethylene glycol 1,000, 0.1 M Barium chloride dihydrate<br>Activity of TREX2 in crystallization condition: Inhibited	Input DNA	DNA in the structure
	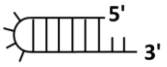	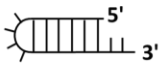
**mTREX2-product complex**		
Input DNA[a]: 5′-AAG**GGCCCTCTTTAGGGCC**TAC-3′<br>Time of growth: 3 month<br>Condition: 0.02 M Nickel(II) chloride hexahydrate, 0.02 M Magnesium chloride hexahydrate, 0.02 M Cadmium chloride hydrate, 0.1 M Sodium acetate trihydrate pH 4.5, 24% w/v Polyethylene glycol monomethyl ether 2,000<br>Activity of TREX2 in crystallization condition: Reduced	Input DNA	DNA in the structure[c]
	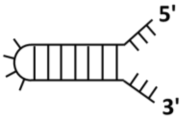	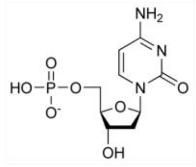

[a] The underline regions are the paired region or stem region of DNA substrates.
[b] The DNA displayed in gray are the disorder regions in the structures.
[c]In the crystal of mTREX2-product complex, the input stem loop DNA was degraded into mononucleotide, deoxycytidine monophosphate.

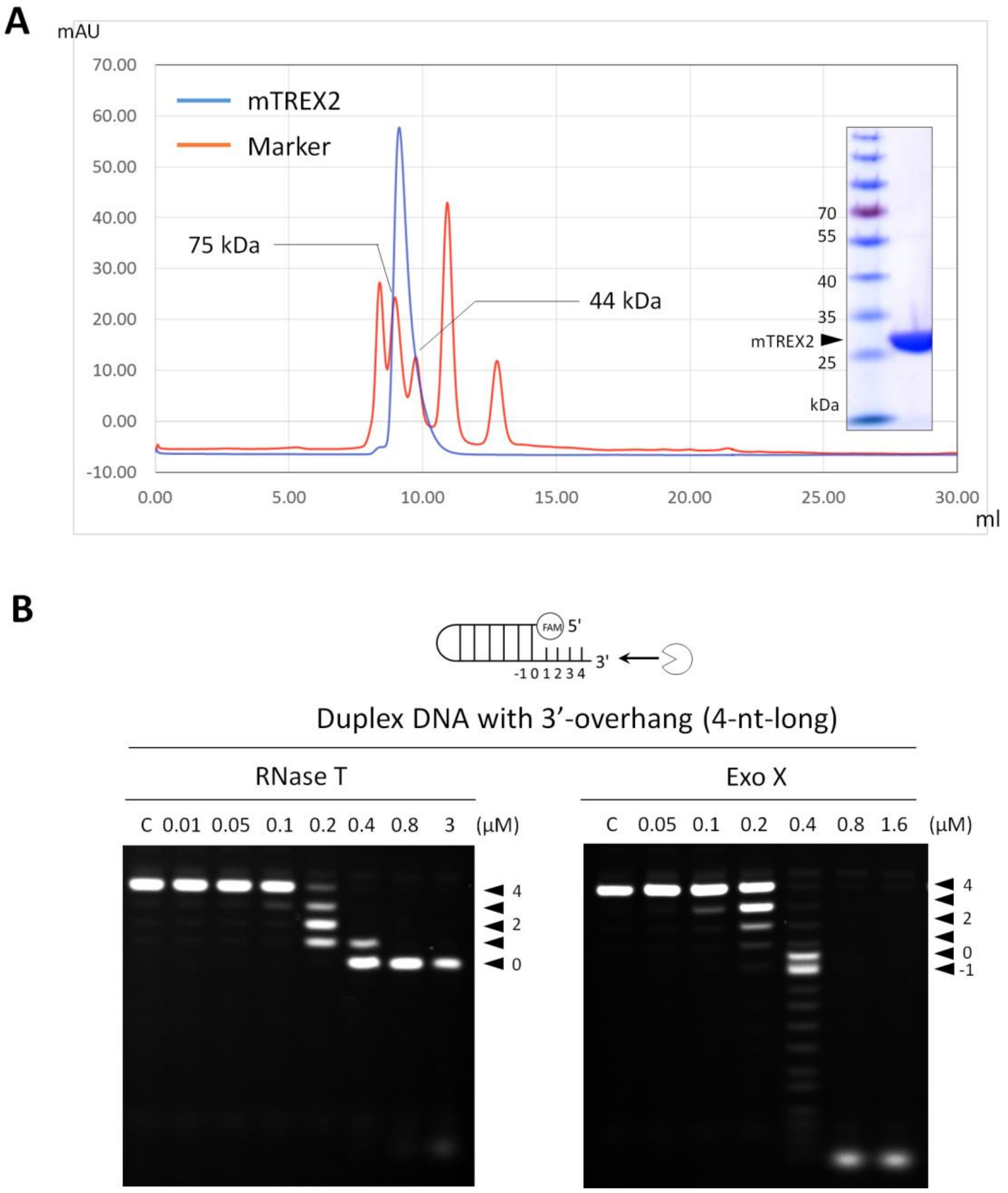

Supplementary Figure 1. The structure of apo-mTREX2. (A) Gel filtration profile of mTREX2. The purified mTREX2 was eluted at a volume corresponding to the apparent molecular weight of ~55 kDa, suggesting that mTREX2 (26.0 kDa) is a homodimer in solution in 150 mM NaCl and 50 mM Tris-HCl at pH 7.0. The indicated markers are conalbumin (75 kDa), and ovalbumin (43 kDa). The purity of the recombinant mTREX2 is shown in SDS-PAGE. (B) Nuclease activities of Exo X and RNase T on digesting duplex DNA with 4-nt-long 3′-overhang. The structural restriction from a double-stranded structure at RNase T is stronger than that at Exo X.

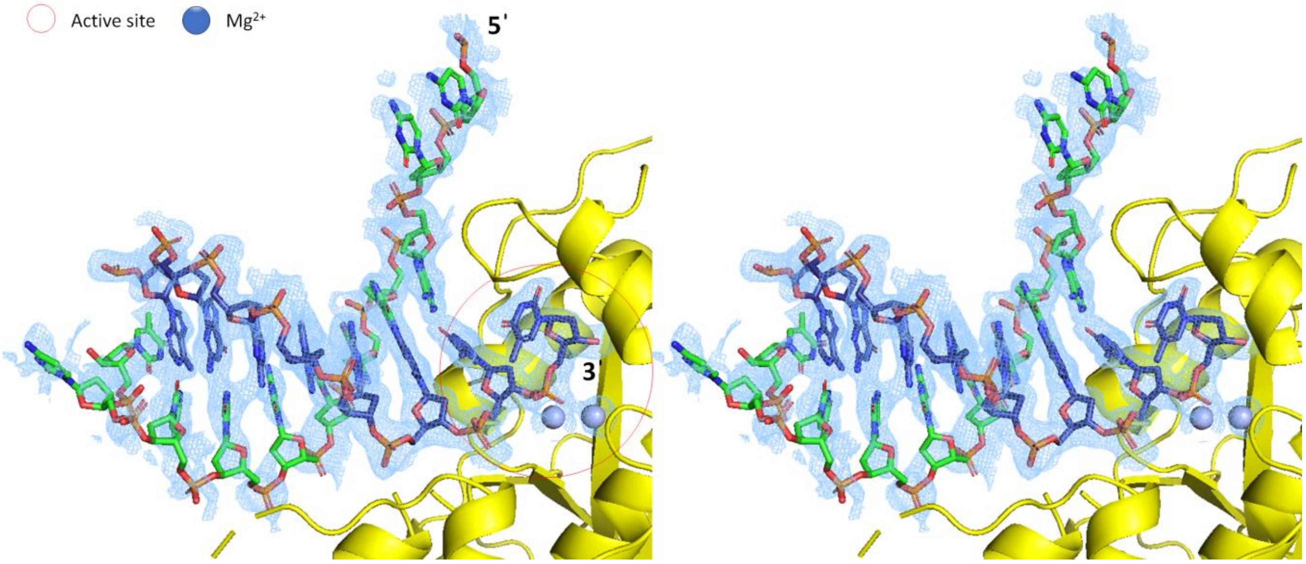

Supplementary Figure 2. The omit map of Y-shaped DNA in mTREX2-Y-shaped DNA complex. The omitted electron density map (blue) is contoured at 1.0 σ. Mg^{2+} molecules are shown in light blue balls. The scissile and non-scissile DNAs are displayed in dark blue and green, respectively.

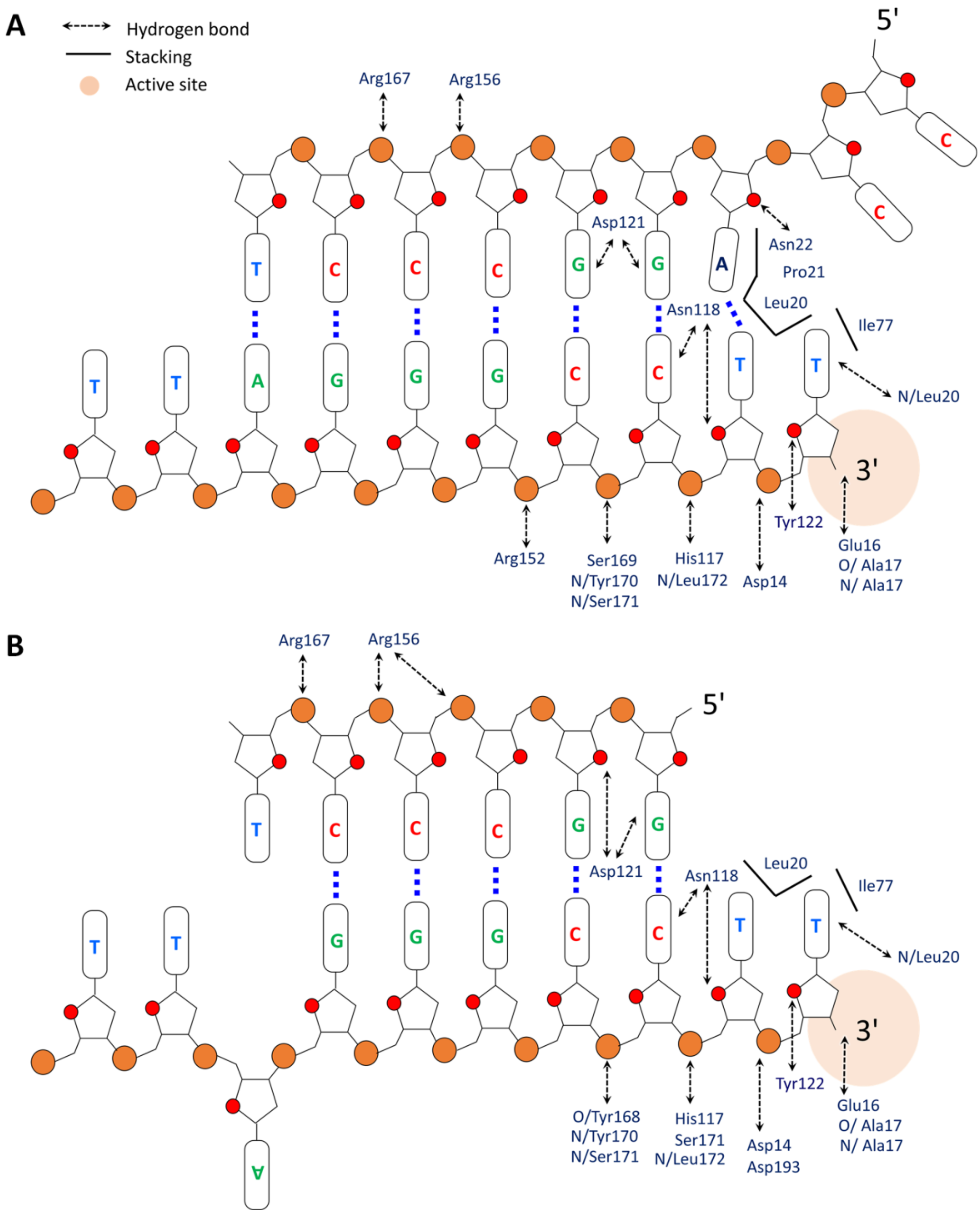

Supplementary Figure 3. Schematic of the interactions between TREX1 and structural DNAs. (A) For the structure of the mTREX2-Y-shaped DNA complex. (B) For the structure of the mTREX2-duplex DNA complex.

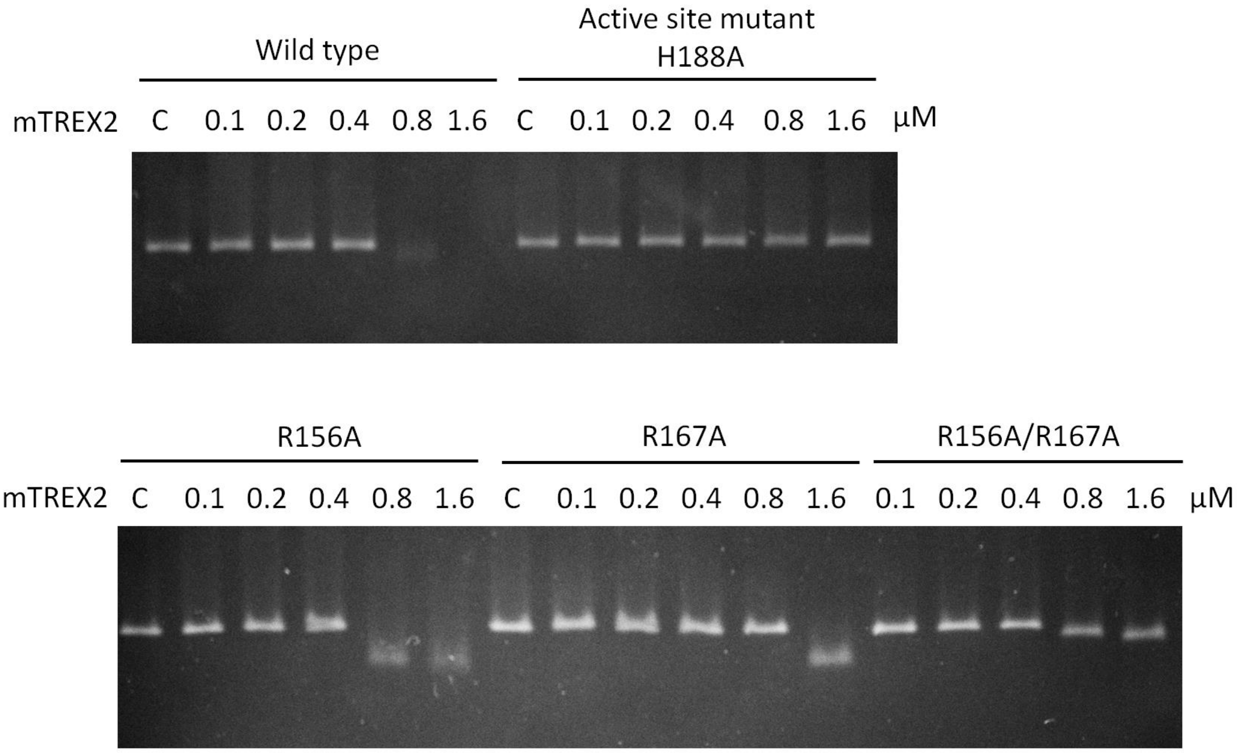

Supplementary Figure 4. The nuclease activity of wild-type mTREX2 and mTREX2 mutants. The nuclease activities of wild-type and mutated mTREX2 in digesting a PCR product (A linear 708 bp dsDNA). The amount of the PCR product was 300ng. The reaction products were then separated on agarose gels and stained with ethidium bromide. The PCR product is fully digested by wild-type mTREX2 at the enzyme concentration of 1.6 μM, but is not fully digested by H188A (active site mutant). Binding site mutants, R156A, R167A and R156A/R167A shown reduced activity on digesting dsDNA at the same enzyme concentration.

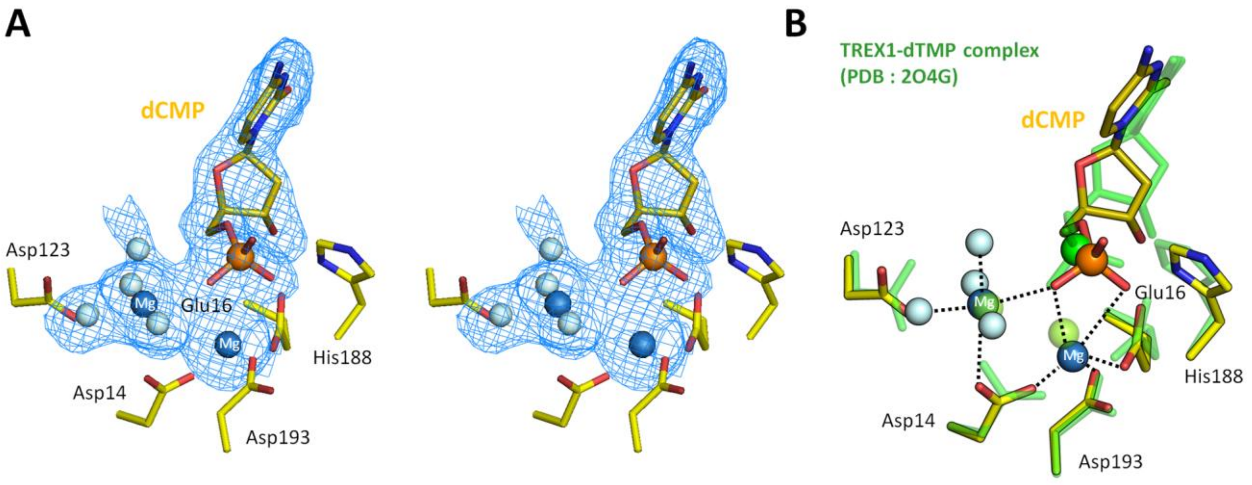

Supplementary Figure 5. The structure and structural comparison of the active site in mTREX2-product complex. (A) The omitted electron density map (blue) is contoured at 2.0 σ. The Mg^{2+} and water molecules are shown as blue and light blue balls. (B) The structural comparison of mTREX2-product complex (yellow) and TREX1-dTMP complex (green). The Mg^{2+} in mTREX2-product complex and TREX1-dTMP complex are colored in blue and green, respectively. Black dotted line is represent the hydrogen bonds between water molecules, Mg^{2+}, dCMP and mTREX2.

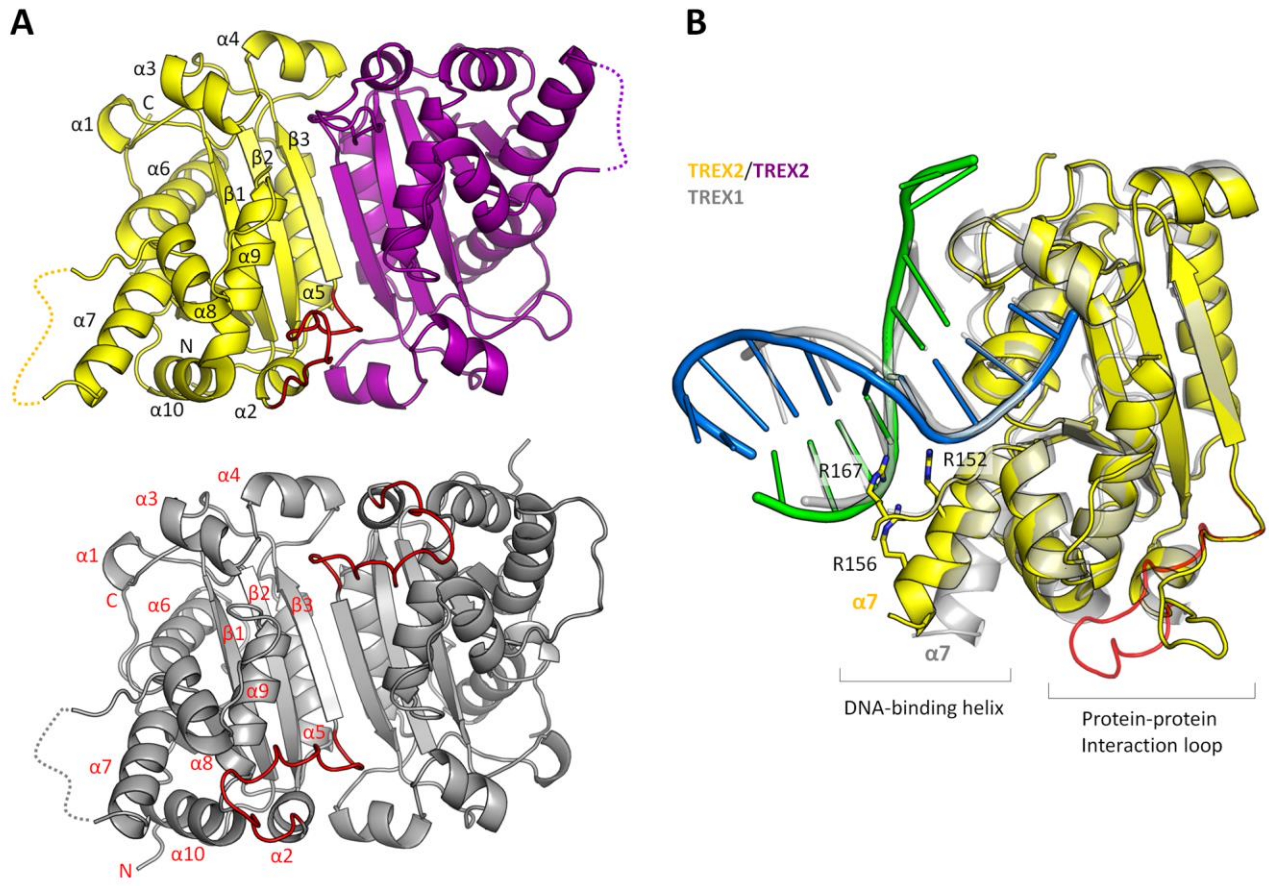

Supplementary Figure 6. The structural comparisons of apo- or DNA bounded TREX2 and TREX1. (A) The distribution of secondary structure in apo-TREX2 and apo-TREX1. The relative position of secondary structures almost identical in TREX2 and TREX1. The protein-protein interacting loops of TREX1 are colored in red. The disordered regions are displayed as dotted lines. (B) The structural difference of TREX2 and TREX1 in DNA binding. Arg152, Arg156 and Arg167 at TREX2 are play the critical roles in binding to duplex DNA.
